# Exploring the feelings of Iranian women of reproductive age about health care seeking behavior: a qualitative study

**DOI:** 10.15171/hpp.2018.09

**Published:** 2018-01-07

**Authors:** Mohammad Ali Morowatisharifabad, Tahereh Rahimi, Tahmineh Farajkhoda, Hossein Fallahzadeh, Siamak Mohebi

**Affiliations:** ^1^Department of Health Education and Promotion, School of Health, Shahid Sadoughi University of Medical Sciences, Yazd, Iran; ^2^Research Center for Nursing and Midwifery Care, School of Nursing and Midwifery, Shahid Sadoughi University of Medical Sciences, Yazd, Iran; ^3^Department of Biostatistics and Epidemiology, Research Center of Prevention and Epidemiology of Non-Communicable Disease, School of Health, Shahid Sadoughi University of Medical Sciences, Yazd, Iran; ^4^Department of Health Education and Promotion, School of Health, Qom University of Medical Sciences, Qom, Iran

**Keywords:** Feelings, Health seeking behavior, Women's health, Qualitative research

## Abstract

**Background:** Despite the important role of feelings in health care seeking behavior (HCSB), this subject has not yet been adequately investigated. HCSB-related feelings begin with the onset of disease symptoms and persist in different forms after treatment. The aim of current study was to explore the feelings that women of reproductive age experience when they seek health care.

**Methods:** In this deductive, qualitative content analysis, participants were selected by purposeful sampling. Semi-structured, in-depth interviews with 17 women of reproductive age and 5 health care staffs in Qom, Iran were carried out until data saturation was achieved. Qualitative data were concurrently analyzed by deductive content analysis, using the Health Promotion Model (HPM). The MAXQDA10 software was used to manage qualitative data analysis.

**Results:** Three main categories were drawn from data to explain the HCSB-related feelings of participants consisting of (1) feeling of inner satisfaction with the treatment with 2 subcategories including "peace of mind" and "feeling alive", (2) multiple roles of fear with 5 subcategories including "fear about the consequences of delay", "fear of having hidden diseases", "fear of unknown experiences", "fear of hearing bad news" and "fear of medical errors" and (3)uncomfortable feelings with 3 subcategories including "feeling uneasy when attending health facility", "feeling embarrassed" and "feeling worthless due to dealing the doctor".

**Conclusion:** This study revealed that the inner feelings of women varied widely, ranging from positive or motivating feelings to negative or inhibitory ones, given their experiences with the formal health care system and the current situation of medical and health services. Highlighting patients’ perceived inner satisfaction and reducing fear and uncomfortable feelings by adopting culture-based practical strategies can enhance women’s HCSB.

## Introduction

There are various definitions for health care seeking behavior (HCSB), but 3 phases are shared by all these definitions: Identifying symptoms; perceiving the nature of illness and seeking care in home at first and then, if needed, seeking health care services, medication outside the home and compliance.^1^ Throughout these phases, many factors may influence individual decision to choose a treatment. In a comprehensive approach, seeking care depends on individual factors (age, gender, detection, interpretation and monitoring of illness symptoms, receiving education about the time of seeking care, income, health beliefs and emotional reaction),^2-4^ interpersonal factors (family support and role of friends and other social networks),^5,6^ community-related factors (gender discrimination and sociocultural status), health care service-related factors (distance to health facilities, perceived quality of care, convenient access to medications and basic laboratory services, health worker attitudes and skills and trust in physicians)^7,8^ and health policies (health insurance-related laws, access to free public health facilities, decentralization in health care, and gender equity in health-related sectors).^9,10^

As with any behavior, HCSB is also associated with feelings. Before, during and after a behavior, positive or negative feelings is encoded in memory as the information and is retrieved when the behavior is repeated.^11^ Although feelings and emotions play a significant role in many health-related decision makings, it cannot be definitely argued that a specific feeling leads to seeking care. Features related to feelings and some other external conditions may lead to unpredictable situations. High levels of fear, for example, can lead to an early attempt to treat a chronic disease such as cancer and acute myocardial infarction; however, the lower levels of fear may alleviate or delay attempts to seek medical care.^12^ In addition to fear, sadness can play a bilateral role when patients choose different types of treatment or screening. Sadness may increase health-related decision-making when medical recommendations are unclear and vice versa. Compared to sadness, happiness and gratitude can improve patient’s trust in health care services. Interestingly, anger also plays the same role. This means that angry patients are more likely to trust health professionals and accept their therapeutic recommendations.^13^ The feelings that patient’s experiences in dealing with health care providers can also affect their selection of treatments. The patient may experience embarrassment, shame or guilt due to the behaviors of health professionals or a specific type of treatment at any step of medical care. The power of these feelings may lead to discontinuation of formal treatment.^14,15^

Feelings toward HCSB are also different between men and women. Gender differences contribute to developing specific feeling about treatment process and therefore welcoming or avoiding health care.^14^This issue is more important for female patients because women contribute greatly to the health of families and the whole community.^16^ As a significant period of women’s life, reproductive age is the time of being faced with unique health challenges that increase the risk of chronic diseases.^17^ Due to the increased exposure to chronic disease risk factors, the significance of timely diagnosis and early treatment increases. However, we are still faced with a great gap in appropriate HCSB and related reasons among women of reproductive age. In addition, to the best of our knowledge, no study has yet been conducted to investigate HCSB-related feelings, while these feelings can be a major contributor to starting or continuing treatment. On the other hand, the sociocultural and environmental factors in the community may contribute to a difference in people’s experiences that can only be investigated by using qualitative approach. This study was conducted, using a qualitative approach, to exploring the feelings of Iranian women of reproductive age about HCSB.

### Theoretical framework

Pender’s Health Promotion Model (HPM) is a comprehensive model for explaining health promoting behaviors. This model consists of 3 categories related to health promotion behaviors: Individual characteristics and experiences; behavior-specific cognitions and affect; and behavioral outcome. Activity-related affect, as a mediator in the HPM, consists of 3 categories: Emotional arousal to the act itself (act related), the self-acting (self-related), and the environment where the action occurs (context related). The resulting feeling can influence whether an individual will exhibit the behavior again or for the long term, or avoid it. Subjective emotional states are developed before, throughout, and after an action, depending on the stimulus features in relation to the behavior of interest. These affective responses can be mild, moderate or strong and are cognitively labeled, stored in memory and relate to subsequent thoughts associated with the behavior. The affect associated with the behavior represents a direct emotional reaction or gut level response to the behavior, which can be positive or negative--fun, enjoyable, delightful or undesirable? Behaviors associated with positive affect can occur again, while those associated with negative affect are more likely to be avoided. Both positive and negative feeling states are induced for certain behaviors. Thus, the relative balance between positive and negative affect before, throughout, and after the behavior should be ascertained.^11^

## Materials and Methods

### Study design 

The study was conducted from May 2016 to January 2017 in Qom, Iran. A qualitative content analysis approach using the HPM was used to investigate the effects of Iranian women’s feelings on their HCSB. Qualitative content analysis can be deductive or inductive. When a qualitative study design is based on exiting theoretical knowledge and the aim of the study is to test a theory, a deductive analysis is adopted. The deductive content analysis consists of 3 main steps, i.e. preparation, organization and reporting results. The preparation step starts with selecting the participants, gathering appropriate data for content analysis, and finding the meanings of the data both as separate units of meaning and as a whole. Based on the current theories and models, in the organization step, a structured or unstructured analysis matrix is formed. All data are frequently reviewed and coded according to the drawn categories. Finally, the analysis process and the results are reported in the reporting step.^18^

### Participants

The participants were 17 women aged 15-49 years and 5 health care staffs (Table 1). Inclusion criteria were being married, Iranian, and of reproductive age, having the ability to communicate appropriately, having at least 1-year work experience for health staffs, and providing informed consent to participate in the study. Exclusion criteria were being pregnant and suffering from the chronic diseases that influence routine HCSB. Purposeful sampling method with maximum variation in terms of age, educational levels, occupational status and residential areas was used to select women of reproductive age and health staffs.

### Data collection

Data were collected using semi-structured, in-depth interviews. Interviews lasted for 35 to 65 minutes on average. After the participants provided consent, the interviews were conducted at the health centers near their homes. The interviews appointed the time of the interviews. To assure privacy, a quiet location near health care providing room was selected as the location of the interview. Before starting the interview, the second author clarified the objective of the study for the participants. All interviews were tape-recorded and continued until data saturation was achieved. Some questions addressed demographic characteristics and an interview guide was developed by the researchers based on the activity-related affect construct described in the HPM. “*What feelings did you experience before, during and after your treatment seeking*?” and “*What feelings prevented or facilitated your referral to a doctor or healthcare provider*?” were the main questions of the research. The follow-up questions were developed to reach more in-depth insights into the experiences of the participants: “*Can you explain your experience with an example*?” and “*Please explain a little more*”. Data saturation was reached after 22 interviews when no further information could be drawn.

### Data analysis

All interviews were recorded using a digital recorder and then transcribed verbatim. The second author coded the transcripts, using unstructured categorization matrix that represented the categories of the HPM. Because in the directed content analysis, the categories are derived from the constructs of the current theories and models,^19^ the activity-related affect construct was considered the main category. Primary coding of the transcripts began and the new categories and sub-categories were drawn according to similarities and differences. The MAXQDA 10 software (VERBI GmbH, Germany, 2010) was used to manage the data analysis.

### Trustworthiness and rigor

The research team maintained its long-term engagement with the subject and findings throughout the study to assure the accuracy and robustness of data, good communication with participants to gain their trust, allocation of sufficient time for participants to express their experiences, and enhancement of the credibility of data. The data dependency capability was achieved by external check and asking some participants to review some transcripts. Sampling of maximum variation (in age, occupation, socioeconomic status and culture) was used to yield greater transferability of data.

## Results

The present study investigated the HCSB-related feelings that the Iranian women of reproductive age experience. Overall, 3 categories and 10 sub-categories were drawn from data that summarizes in Table 2.

### 1. Feeling of inner satisfaction with the treatment

Although most participants reported that they experienced negative feelings as they were seeking medical care, a number of participants reported experiencing positive feelings which arose from their feeling of inner satisfaction with the treatment. Feeling alive and peace of mind after referring to a doctor or other health professionals during an illness, encouraged the women to seek further care in the formal health care system.

*1.1. Peace of mind*

This feeling was due to the calmness and confidence that participants experienced while consulting with the doctor about the cause of their disease and treatment method. When they were assured that illness symptoms were not life-threatening or serious, they felt comfortable. They also reported that they were sometimes confused and bewildered due to coping with illness symptoms, which imposed nervous pressure on them. When the doctor clarified the issue with additional information, this mental pressure was relieved so that they felt relaxed.

“*When I took medicine on myself, I’m not sure* [that] *I’d get well. Even though I’m very much confident about herbal drugs, when I use my herbal drug, I have some doubts whether it can make me good, but* [when]* I go to the doctor’s office, I feel comfortable that I’d get well then because my treatment has been based on the doctor’s opinion*” (Participant 2).

“*For a while, I felt that my hand was burning, my foot was burning, as if a thousand knits were being simultaneously snapped on my fingertip, this was much annoying for me*, *but I couldn’t find out what my problem was, every one gave an idea, some* [people] *said* [that]* you had diabetes, some* [people] *said* [that] *it was due to your nerves. I referred to the doctor and had a test. The doctor said: Don’t worry, the cause of* [feeling] *of your hand and foot being repeatedly punched by needle or your immediately getting angry is that your blood hemoglobin was low and you should treat your anemia. When I heard these* [words], *I felt relaxed very much because I was really confused*” (Participant 5).

*1.2. Feeling alive*

The other feeling that participants, especially those who had passed a hard-to-treat disease, reported was a mental feeling of being able to outlive after successful treatment. The participant no. 12 was a 40-year-old woman who had survived breast cancer. She described her feeling as follows: “*I passed hard days. After you get rid of a dangerous disease, you’ll feel your life with all your existence. You thank God for every moment of being alive because you really understand being alive. As you get closer to death, this feeling gets stronger in your existence. That is, till it doesn’t happen to oneself, he won’t understand what I say*”.

Another participant said: “*I have little child, I’m wishing that they’ve grown up and I’ll see my children’s wedding, being healthy gives me more hope for long longevity, as you feel that you can see the future is a very good feeling*” (Participant 17).

### 2. Multiple roles of fear

Fear was one of the prominent feelings that participants experienced. Although fear can have a negative nature, it can also serve as both inhibitor and facilitator of taking action to be treated. Many of the participants prioritized medical treatment due to fear of illness. They also reported that they were afraid of seeking care if they needed to use medical diagnostic procedures about which they did not have any information. Fear of hearing bad news or detecting hidden diseases during the treatment also brought about some degrees of stress and anxiety. Ultimately, the fear of medical errors led to delayed seeking of care.

*2. 1. Fear about the consequences of delay*

Some participants reported that when unpleasant symptoms appeared, they conducted a mental evaluation of the terrible consequences due to lack of referring. This fear forced them to refer to the health staffs. Fear of having a severe illness or cancer, fear of spreading the disease to the whole body, fear of losing organs, or fear of death as a result of lack of referring to the doctor were some of the feelings that can be included in this sub-category.

“*In the medical programs of the TV, I heard that any disease could have bad consequences if it is not treated. It scares me, sometimes I think on myself if I refer late, something may happen at once, I may get cancer at once*” (Participant 1).

“*I know* [that] *all gynaecological diseases are related to the uterine. It’s said that all diseases enter from woman’s uterine. When vaginal infection remains, it grows gradually and turns into a mass, then it spreads out to the rest of her body and finally kills her, thinking of all these is very scary”*( Participant 3).

*2.2. Fear of having hidden diseases*

Fear of having hidden diseases also led to taking action to be treated and undergo screening and laboratory tests.

“*I’m a stressful woman, If I have a disease in my body that manifests itself late, I’m afraid of it very much. I wish it manifests itself sooner so that I find out* [it]*. Even if it’ll be clear that it’s not been anything but I’ve got suspicious, definitely I’ll refer to the doctor*” (Participant 14).

“*My husband works in hospital, I’m always afraid he’s got hepatitis or AIDS and I’ve also got from him and I myself don’t know. That’s why I try to have a blood test each year so that if I’ve got such disease, I would find out*” (Participant 9).

*2.3. Fear of unknown experiences*

When the participants used a medical diagnosis for the first time or did not have any information on their own treatment, they experienced fear. The participant no. 4 described her feelings when he had to undergo endoscopy for the first time: “*I was sitting back of the doctor’s room, inside the room for a woman they were conducting endoscopy. She cried so much, and had such a gagging voice that I was going to have heart attack from fear there. I would say to myself, God, what are they doing there?*”

Another participant experienced such feelings years ago when a doctor asked him to conduct MRI [Magnetic Resonance Imaging] for diagnosis of the cause of her back pain: “*Doctor said to me that you should go* [to have] *an MRI, I remember that I was afraid* [of it] *very much*. *I had taken blood test, urine* [test]*, and or sonography several times but not MRI, that time was not similar to now when everyone knows how it is, I knew nothing about it, I couldn’t say its name at all”* (Participant 15).

*2.4. Fear of hearing bad news*

Fear of hearing bad news was one of the reasons for which the participants did not refer to doctor. Many of the participants experienced delaying seeking health care for different diseases because of being afraid of confirming diagnosis of serious illness and the need for being hospitalized.

“*Now you get a common headache, you take a sedative on yourself* [and] *get well but* [when] *you refer to the doctor, it is possible that they don’t tell you good things, one says* [that] *it’s from migraine, one says* [that] *it’s from your brain, he says* [that] *it’s from cancer. Doctors just bring fear and stress for one’s soul with their words. This by itself causes thousands of other illnesses*” (Participant 16).

“*You know my mom had anemia* [and] *I’ve got* [anemia] *too. Her thyroid had problem, my* [thyroid] *got problematic too, now whenever I refer to gynecologist, these fears are with me that the doctor may tell that you too have fibroma* they developed feelings of fear* and should undergo surgery … when I fetched sonography to show* [it] *to the doctor I recite many Salavats so that the doctor wouldn’t say* [that]* your test hasn’t been good*” (Participant 7).

*2.5. Fear of medical errors*

Some participants felt certain degrees of fear due to having experienced medical errors or knowing the people that have experienced such errors. The most frequent cause of this type of fear, was to acquire infectious diseases due to contact with infected medical equipment and medical errors in surgery.

“*A relative of ours referred to the doctor* [and]* said* [that]* because the doctor didn’t disinfect the endoscopy tube, he’s got hepatitis. I’m also now afraid I refer for endoscopy or for example refer to dentistry. I’m always worried [and] wonder if I’d get hepatitis from these devices of them*” (Participant 2).

“*I have problem with my tonsils. When I was young, I should’ve been operated. Now when I catch a cold, I’m annoyed very much. The doctor now says* [that] *we do surgery but it’s also possible that your face or otherwise gets problematic, we can’t assure* [you that] *no problem would happen to you, for being afraid of that they may cause a catastrophe for me, I wouldn’t refer for surgery*” (Participant 9).

### 3. Uncomfortable feeling

Uncomfortable feeling consisted of 3 sub-categories: feeling uneasy due to treatment location, feeling embarrassed and feeling valueless due to dealing with a doctor.

*3.1. Feeling uneasy when attending health facility*

When the participants referred to public hospitals for treatment, they experienced uncomfortable feelings. The lack of privacy, the space infused with the patients’ suffering and pain, anxiety due to presence in the hospital and the remembrance of the loss of their loved ones made it difficult for them to tolerate the space. Many of the participants experienced uncomfortable feelings due to presence of other patients while visiting the doctor in the public hospitals.

“*In the public hospitals, a number of patients are sent inside* [the doctor’s office]. *One wanna say something* [to the doctor] *that he may not be willing to say in the presence of others. For example, when I referred for my breast mass problem, I encountered our neighbor, we’re visited simultaneously, when she found out that I have problem, the entire neighborhood found out.*” (Participant12).

Some of them experienced uncomfortable feelings due to physical changes such as heart: “*As soon as I enter the hospital, I get a heart beat. Sometimes I go as patient caregiver,* [as if]* my heart is gonna get out of my chest so severely that I wish to leave there as soon as possible, in our hospitals you go on your feet but then your coming back is with God*[‘s will]” (Participant 7).

*3.2. Feeling embarrassed*

The participants reported that they were more comfortable with female physicians, and that if the doctor was male, they felt more embarrassed: “*To female doctors, I tell my problems much more easily, sometimes when I refer to a male doctor, he asks me the questions that I feel embarrassed to answer to, for example I referred for urinary tract infection they asked* [questions] *about* [my]* sexual intercourse with my husband so one feels embarrassed*” (Participant 9).

In addition, the perceptions regarding the type of the disease and the physician’s encounter that they had previously experienced induced feeling of embarrassment. The participant no. 10 who had presented with abnormal discharge from her breast since approximately one year ago described her feeling that prevented her from referring to the doctor:

*“I feel embarrassed to refer to the doctor and say* [that] *purulent discharge gets out of my breast, I used to say to myself* [that] *no one has this disease, perhaps the doctor will ask what did you do that this condition happened to you? Perhaps he’ll say* [that] *you didn’t observe health so much that the infection has entered from your breast’s nipple and discharges black dirt, I didn’t know what to say*.”

*3.3. Feeling worthless due to dealing the doctor*

A number of participants reported that they felt worthless due to previous dealing with the doctor. Some of them were verbally humiliated by the physicians at examinations. Some others felt that the doctors did not see the patients well or ignored them, and therefore had uncomfortable feelings during treatment.

*“The behaviors of some of them are too bad to talk to them, they don’t even reply to your hello. As if they’ve been thrown from elephant’s nose. They didn’t pay attention to you and do not treat you as a respectable person. Well, one is not comfortable with such doctors*” (Participant 5).

“[When being] *with doctors, I don’t have a good feeling. The doctors don’t like us. They don’t look at us as human. [At] that time you feel [that] you’re low. They look at us from the top, as if he himself or his family have never been sick or will [never] get ill*” (Participant 13).

## Discussion

The current study for the first time investigated the feelings of women of reproductive age in Qom, Iran as they were seeking health care. A patient’s feelings represent a key factor for his/her taking decision to be treated or not. While negative feelings can decrease treatment adherence, positive feelings are more likely to serve as a motivating factor for initiating or continuing treatment.^20^ Peace of mind and feeling alive were some of the positive feelings that our participants experienced after treatment. Rubin et al argued that willingness to resolve concerns and build confidence and peace of mind was one of the reasons that led swine flu patients into receiving medical counseling.^21^The study of Moridi et al on meanings of health from diabetic patients’ perspectives, showed that the patients could not feel healthy without peace of mind.^22^Consistent with the current study, feeling alive following adherence to treatment among the patients has already been reported by some researchers.^23,24^ Having experienced such feelings can increase the likelihood of seeking health care from formal sources of health care. Feeling of inner satisfaction with the treatment indeed acts as a reward for doing a behavior at a later time. Health professionals can increase the likelihood of continuing appropriate health promoting behaviors among women by reminding them of the positive feelings they have already experienced.

Fear was one of the key feelings that participants had experienced since observing illness symptoms until initiating treatment. While fearing the consequences of delay and hidden illnesses encouraged them to visit health care staffs, they reported fear of unknown experiences, medical errors and hearing bad news to be emotional barriers to delaying it. Consistent with our study, Unger-Saldana et al reported that feeling of fear can lead to a dual approach to seeking health care in women.^25^ The fear about severe and irreversible consequences in our participants accelerated seeking health care. In some studies, such consequences have been found to contribute to initiating treatment. For example, fear of death has been reported, by de Nooijer et al, to be the most prominent consequence of delay in referring to physicians as a motivating force for seeking medical care.^26^ It seems that enhancing knowledge about the symptoms of hidden diseases and the consequences of deferring treatment through various educational media can bring about some degrees of fear in women, which are sufficient to convince them of taking action to be treated. The results of a survey of adults in London indicated that feeling of fear in people who experienced at least one out of 10 symptoms of cancer in the past 3 months, somehow influenced their HCSB. Some participants sought care because of fearing the consequences of cancer symptoms and perceiving cancer development due to these symptoms. Besides that, fear due to being aware of having cancer in some women does not allow them to consult their doctors about their problems.^27^ A study conducted by the National Cancer Institute’s 2008 Health Information National Trends Survey (HINTS), has revealed that fear of hearing bad news such as confirmed diagnosis of disease or exacerbation of a previously diagnosed condition is one of the main reasons for avoiding medical care among American adults.^28^ Learning skills for communicating with patients, especially how to deal with patients’ emotional reactions to bad news, helps clinicians to lead patients into developing positive attitudes toward disclosure of disease-related news.^29^

Lack of information about disease and its treatment is often associated with certain degrees of fear and concern. The study of Peek et al demonstrated that fear due to lack of information about the procedure to be performed during a mammography process was one of the reasons for not referring for screening tests such as screening mammogram.^30^ Health care providers are required to help patients reduce their levels of fear due to ambiguities regarding treatment process by clarifying all steps of treatment and particularly the way in which clinical testing is carried out.

In our study, some women reported avoiding diagnostic test or medical care due to fear of possible medical errors throughout treatment process. Taking decision to use formal health care systems will become more common if health care services are high quality and error-free in addition to being based on patient preferences.^31^ Because patients’ perceptions of medical errors or, in their own words, medicine-associated complications influence their satisfaction and consequently treatment adherence, they should be taken into account even if they do not reflect an undesirable situation in reality.^32^ Along with compliance with the international standards for reducing medical errors, patients should be sufficiently assured that attempts are being made to deliver high quality care to them.

The current study also showed uncomfortable feelings that women experienced as treatment began. The participants in our study reported to experience uneasy feelings as they attended health facilities and felt embarrassed and worthless in dealing with health care staff. Some researchers have reported that as the patients are seeking health care, they may experience uncomfortable feelings due to certain issues such as physical examination, procedures and conditions of performing health care services, and talking with health care staff about certain symptoms of disease.^28,33,34^ A qualitative study in public hospitals in Iran showed that the female patients’ uncomfortable feelings were mainly due to inappropriate hospital attire and the presence of male staff, visitors and patient in the room or other care units. Some participants also reported that they felt that hospital staff do not like and respect them.^35^ Nikbakht Nasrabadi et al have argued that seeking information about problems related to the genitalia and sexual health is the main reason for feelings of embarrassment among Iranian women compelling them to ignore the problem or to seek health care from non-formal sources of health care.^36^ Since maintaining the value and dignity of a patient is one of his/her fundamental rights, it is better, in any community, to provide health care services in accordance with the sociocultural and religious backgrounds of individuals. Optimizing the environments of public health facilities with respect to privacy and physician and patient gender concordance, strengthening the communication skills of health care providers to deal with patients in an unbiased manner, and avoiding humiliating or neglecting them can reduce uneasy feelings in patients. If we seek to have patients use the health care of formal health facilities more frequently, health professionals need to modify their communication with patients in addition to encouraging them to maintain positive feelings and reduce inhibitory ones. Health professionals can improve patients’ inner feelings if they put themselves in their shoes and look at the situation from their viewpoints. Public health facilities, as the most frequently referred health care centers, also need to reform their physical structure and improve the quality of the health care services they provide.

A limitation of our study was enrollment of the participants only from urban areas. Because geographical and sociocultural contexts of rural and urban areas are different, it seems that valuable data on rural women can also be drawn. Therefore, additional studies with subjects of different ages are recommended to obtain more comprehensive information on Iranian women’s experiences with HCSB.

## Conclusion

The results of the current study provided novel information about the feelings of women before, during and after seeking health care services. Although this issue has been less frequently studied comprehensively, the determinative role of feelings in starting and continuing HCSB cannot be disregarded. Enhancing patients’ inner satisfaction toward formal health services and reducing fear and other uncomfortable feelings by adopting sociocultural-based practical interventions can improve women’s HCSB.

## Ethical approval

Ethical approval for this study was obtained from the Institutional Review Board at School of Public Health, Shahid Sadoughi University of Medical Sciences, Yazd, Iran (IR.SSU.SPH.REC.1394.89). The participants provided written informed consent before each interview. Participation in this study was voluntary, and the women were assured that they could withdraw from the study at any time without any effect on the quality of free health care services they received.

## Competing interests

The authors declare no conflict of interest.

## Authors’ contributions

This study was a part of PhD thesis of the second author (TR). TR and MAM developed the framework of the study. Qualitative data collection and initial analysis was conduct by TR. MAM, TF, HF and SM contributed to all stages of the research, final analysis and editing, and commented on the paper. All authors read and approved the final version of manuscript.

## Acknowledgements

This study was financially supported by Shahid Sadoughi University of Medical Sciences, Yazd, Iran. The authors wish to appreciate all women for their participation in this study.

**Table 1:** Participants′ characteristics

**Variables**	**Reproductive age women**	**Health care staffs**
Age		
Range	17-47	26-45
Mean (SD)	31.91(56)	37.60 (7.43)
Educational status		
Illiterate	1(5.88)	-
≤12 grade	11(64.70)	-
≥12 grade	5(29.42)	5 (100)
Occupational status		
Housewife	11 (64.70)	-
Employee	4 (23.54)	5 (100)
Self-employed	1(5.88)	-
Laborer	1(5.88)	-
Number of children		
Range	1-6	0-4
Mean (SD)	4.25 (0.12)	2 (1.58)
Health Insurance coverage		
Yes	15 (88.2)	5 (100)
No	2 (11.8)	-

**Table 2:** Categories and subcategories of HCSB in reproductive age women

**Construct**	**Categories**	**Subcategories**
HCSB-related feelings	Feeling of inner satisfaction with the treatment	1) Peace of mind
		2) Feeling alive
	Multiple roles of fear	1) Fear about the consequences of delay
		2) Fear of having hidden diseases
		3) Fear of unknown experiences
		4) Fear of hearing bad news
		5) Fear of medical errors
	Uncomfortable feelings	1) Feeling uneasy when attending health facility
		2) Feeling embarrassed
		3) Feeling worthless due to dealing the doctor

Abbreviation: HCSB, health care seeking behavior.
